# Optimizing non-invasive preimplantation genetic testing: investigating culture conditions, sample collection, and IVF treatment for improved non-invasive PGT-A results

**DOI:** 10.1007/s10815-023-03015-3

**Published:** 2024-01-06

**Authors:** Judy F. C. Chow, Kevin K. W. Lam, Heidi H. Y. Cheng, Shui Fan Lai, William S. B. Yeung, Ernest H. Y. Ng

**Affiliations:** 1 Department of Obstetrics and Gynaecology, School of Clinical Medicine, LKS Faculty of Medicine, The University of Hong Kong, Hong Kong, China; 2 Department of Obstetrics and Gynaecology, Queen Mary Hospital, Hong Kong, China; 3 Department of Obstetrics and Gynaecology, Kwong Wah Hospital, Hong Kong, China; 4 Shenzhen Key Laboratory of Fertility Regulation, The University of Hong Kong-Shenzhen Hospital, Shenzhen, China

**Keywords:** Non-invasive, PGT-A, Spent culture media, Aneuploidy, niPGT-A

## Abstract

**Purpose:**

This study aimed to optimize the non-invasive preimplantation genetic testing for aneuploidy (niPGT-A) in the laboratory by comparing two collection timing of the spent culture medium (SCM), two embryo rinsing protocols, and the use of conventional insemination instead of intracytoplasmic sperm injection (ICSI).

**Methods:**

Results of two embryo rinsing methods (one-step vs sequential) and SCM collected on day 5 vs day 6 after retrieval were compared against trophectoderm (TE) biopsies as reference. Results from day 6 SCM in cycles fertilized by conventional insemination were compared with PGT-A using ICSI.

**Results:**

The rate of concordance was higher in day 6 samples than in day 5 samples when the sequential method was used, in terms of total concordance (TC; day 6 vs day 5: 85.0% vs 60.0%, *p* = 0.0228), total concordance with same sex (TCS, 82.5% vs 28,0%, *p* < 0.0001), and full concordance with same sex (FCS, 62.5% vs 24.0%, *p* = 0.0025). The sequential method significantly out-performed the one-step method when SCM were collected on day 6 (sequential vs one-step, TC: 85.0% vs 64.5%, *p* = 0.0449; TCS: 82.5% vs 54.8%, *p* = 0.0113; FCS: 62.5% vs 25.8%, *p* = 0.0021). There was no significant difference in niPGT-A results between cycles fertilized by the conventional insemination and ICSI.

**Conclusion:**

We have shown a higher concordance rate when SCM was collected on day 6 and the embryos were rinsed in a sequential manner. Comparable results of niPGT-A when oocytes were fertilized by conventional insemination or ICSI. These optimization steps are important prior to commencement of a randomized trial in niPGT-A.

**Supplementary Information:**

The online version contains supplementary material available at 10.1007/s10815-023-03015-3.

## Introduction

Aneuploidy rate of embryos increases exponentially with advancing maternal age and is a major reason for failure, pregnancy loss, and congenital anomalies following both natural conception and pregnancies conceived by in vitro fertilization (IVF) [1–5]. Preimplantation genetic testing for aneuploidies (PGT-A) selects euploid embryo for transfer and thus may increase the live birth rates and decrease miscarriage rates per transfer. PGT-A improves embryo selection and thus decreases time to pregnancy [6]. Next-generation sequencing (NGS) is currently the most commonly used platform for PGT-A [7, 8].

Reduction in futile transfer and pregnancy loss can reduce the psychological burden experienced by infertile women [9]. Early randomized trials showed that PGT-A improved clinical pregnancy rate [10, 11], or implantation rate [12]. However, subsequent randomized trials failed to demonstrate a benefit of PGT-A on improving overall pregnancy rate [13], live birth rate in young infertile women [14], or cumulative live birth rate in young women [15]. A recent systematic review and network meta analyses of 11 randomized trials found that PGT-A only improved live-birth rates in women with advanced age (> 35 years), but not in the general population [16].

At present trophectoderm biopsy is performed on blastocysts on day 5/6/7 after oocyte retrieval to obtain 5–10 cells for genetic analysis [17, 18]. It is an invasive procedure and requires well-trained staff and additional instrument such as laser equipment. Although trophectoderm biopsy is less detrimental to the implantation of embryos when compared with cleavage stage biopsy [19], the implantation potential may still be negatively affected by the number of cells biopsied [20]. It is possible that the detrimental effect of trophectoderm biopsy nullifies the benefit of PGT-A. Therefore, a non-invasive approach for PGT-A is desirable.

Cell free DNA (cfDNA) is detectable in spent culture media (SCM) of human embryos and has been proposed to be a potential marker for embryo assessment [21]. This opens the possibility of non-invasive PGT-A (niPGT-A). Collection of SCM requires neither highly trained staff nor expensive equipment, therefore making niPGT-A more accessible to IVF laboratories.

Several studies compared niPGT-A results on SCM with the corresponding trophectoderm biopsy on vitrified embryos and reported a high amplification rate ranging from 80.4 to 100% [22–24], and a concordance rate up to 93.8% [24]. However, false negative results were reported, which were attributed to maternal cells/DNA contamination [25]. Single-nucleotide polymorphism (SNP) study revealed high variability in percentage of embryonic DNA observed in SCM, ranging from 0 to 100% [26]. The contamination with maternal genetic materials becomes prominent in the media harvesting in the early window (days 1–3) of embryo culture [26, 27]. Optimization in embryo culture workflow is urgently needed to maximize the representation of embryonic genome in SCM.

Furthermore, all relevant studies on niPGT-A focus on amplification success and concordance between SCM and trophectoderm biopsy in PGT cycles, in which oocytes were denuded by enzymatic and mechanical removal of surrounding cumulus cells before fertilized by single sperm injection. The efficacy of niPGT-A on embryos fertilized by conventional insemination remains uncertain.

A large multi-centre randomised trial is urgently needed to confirm its efficacy in embryo selection during IVF in terms of live birth and miscarriage rates. Optimizing the niPGT-A protocol in the laboratory is of importance prior to commencement of the clinical trial.

This study aimed to optimize niPGT-A by focusing on the embryo rinsing protocols and the collection timing of the SCM. Efficacy of niPGT-A on embryos fertilized by conventional insemination instead of intracytoplasmic sperm injection (ICSI) was investigated. Finally, the concordance rate of two IVF centres was also evaluated when the optimized niPGT-A protocol was applied.

## Materials and methods

Women undergoing IVF cycle in the Centre of Assisted Reproduction and Embryology, The University of Hong Kong, Queen Mary Hospital (QMH), and Kwong Wah Hospital (KWH) between February 2020 and March 2021 were included in this study. All women agreed to donate the spent culture media and the non-utilizable embryos for research. Ovarian stimulation, oocyte retrieval, fertilization by conventional insemination or ICSI, embryo grading, and culture were performed as previously described [18].

### SCM in the early window (days 1–3) of embryo culture

Fertilization medium was collected on day 1 (*n* = 14). Cumulus cells and sperms in the medium were removed by centrifugation at 10,000 × g for 10 min, and the supernatant was collected. Normally fertilized embryos by conventional insemination were cultured individually in a monophasic medium (G-TL, Vitrolife) in 12 µL micro-droplets, and the culture medium was replenished in the morning on day 3. The embryos were rinsed in a 500-µL G-TL culture drop before transferring to a fresh micro-droplet for further culture (One-step rinsing). Embryos that did not develop into utilizable blastocysts but reaching morula stage or later were collected. Utilizable blastocysts were those with an expansion score of 3 or above, inner cell mass, and/or trophectoderm grades of B or above according to the Gardner grading system [28]. Whole embryos were collected on day 6, rinsed in 1% PVP in PBS, transferred into PCR tube, and stored at − 80 °C until use.

Eight microliters of SCM of the same embryo (*n* = 11) were collected sequentially on day 3 and day 6 after oocyte retrieval using molecular grade filter pipette tips. A new pipette tip was used for each SCM sample. Medium incubated in parallel under the same conditions but without embryo contact was collected on day 6 as control. All the samples were stored at − 80 °C until use. Results on SCM were compared whole embryos.

### Comparison of embryo rinsing methods

Two embryo rinsing methods, one-step rinsing vs sequential rinsing, were compared in the study. The one-step rinsing method (*n* = 65) involved rinsing the embryos in a 500-µL G-TL culture drop on day 3 before transferring to a fresh micro-droplet for further culture. In the sequential rinsing method (*n* = 88), embryos in group were rinsed in five 500 µL G-TL culture drops before transferring to fresh culture micro-droplets for culture till day 5 or 6. For both rinsing methods, embryos were rinsed in a group of at most three embryos at a time. Results on SCM were compared with trophectoderm (TE) biopsy.

Depending on the development of blastocysts, TE biopsy was performed on day 5 or day 6 after oocyte retrieval. In brief, zona drilling was performed on day 4 using a Saturn 5 laser system (Research Instruments). The biopsy was performed by aspiration of five to ten cells from the trophectoderm using a biopsy needle with the aid of a laser.

### NiPGT-A in IVF cycles using conventional insemination vs intracytoplasmic sperm injection (ICSI)

Whole embryos (*n* = 101) and TE biopsies (*n* = 47) were used as reference in the conventional insemination group and the ICSI group, respectively. The sequential rinsing method was applied, and SCM was collected on day 6.

### Comparison between two IVF centers

Normally fertilized embryos by conventional insemination that did not develop into utilizable blastocysts but reaching morula stage or later were collected in QMH (*n* = 35) and KWH (*n* = 66). Sequential rinsing method was applied, and SCM were collected on day 6. Results on SCM were compared whole embryos.

### PGT-A on trophectoderm biopsy and whole embryos

NGS analysis on trophectoderm biopsy and whole embryos was performed by the VeriSeq-PGS MiSeq (Illumina, UK) as previously described [29]. In this study, classification of aneuploidy in trophectoderm biopsy and whole embryos was determined by CNV values. A CNV value ≤ 1.2 was considered as loss, and a CNV value ≥ 2.8 was considered as gain. Whole chromosome aneuploidy was automatically called by the BlueFuse Multi Software (v4.5) when the CNV value met the cutoffs (1.2 and 2.8). All segmental aneuploidies were manually called around the breakpoints (> 20 Mb). Mosaic aneuploidy was not called in the study because the NI-PGT kit has not been validated for reporting mosaicism.

### Non-invasive PGT

cfDNA in SCM was analyzed by the NI-PGT kit (PG-Seq Rapid Non-Invasive PGT kit, PerkinElmer) following the manufacturer’s protocol. In brief, the kit followed a single-tube workflow, two-step PCR to whole genome amplification of the DNA in the SCM and then attached indexes and sequence-specific adapters to template DNA, resulting in sequencing ready samples.

After purification, equal molar concentration of indexed DNA from 96 samples were pooled and sequenced on a MiSeq system (Illumina) at 1- × 75-bp read length. On-board secondary analysis was performed automatically by the MiSeq Reporter (Illumina) followed by the PG-Find Software with default setting (v1.0, PerkinElmer). Reads aligning to anomalous, unstructured, and highly repetitive sequences were filtered from the analysis. A target bin size of 1000 Kb was used. All genomic positions were referred to the human genome build NCBI 37. Mosaic aneuploidy was not called in the study because the NI-PGT kit has not been validated for reporting mosaicism.

Classification of whole chromosome aneuploidies and segmental aneuploidies (> 20 Mb) in SCM was determined by the default settings on CNV (copy number variation) values. A CNV value ≥ 2.7 was considered as gain, while a CNV value ≤ 1.3 was considered as loss.

To analyze the reliability of SCM, we investigated the concordance between TE biopsies and SCM in terms of aneuploidy detection. Samples with results were classified into four categories.

1. Full concordance: Both TE and SCM samples were aneuploidy on the same chromosomes or both samples were euploid.
2. Total concordance: both samples were aneuploid with one of the following observations:
  - the results were complementary to each other in terms of gain or loss on the same chromosome (aneuploidy-complementary)
  - the same aneuploidy was detected on at least one chromosome
  - the aneuploidies detected on completely different chromosomes
3. Discordance: one sample was aneuploid while the other was euploid.
4. Non-informative result: SCM showed either no conclusive result when NGS quality score > 2.0, or chaotic results when the number of aneuploidy detected ≥ 5.

### Statistical analysis

Chi-square test was used for categorical data and Fisher exact test was performed when the sample size was small. For comparison of quantitative values for post-amplification DNA concentration, ANOVA with multiple comparisons (Tukey’s HSD) was performed. All statistical analyses were performed with R (version 4.03). *P* < 0.05 was considered as statistically significant.

## Results

### Maternal cell contamination in the early window (days 1–3) of embryo culture

**Fig. 1:**
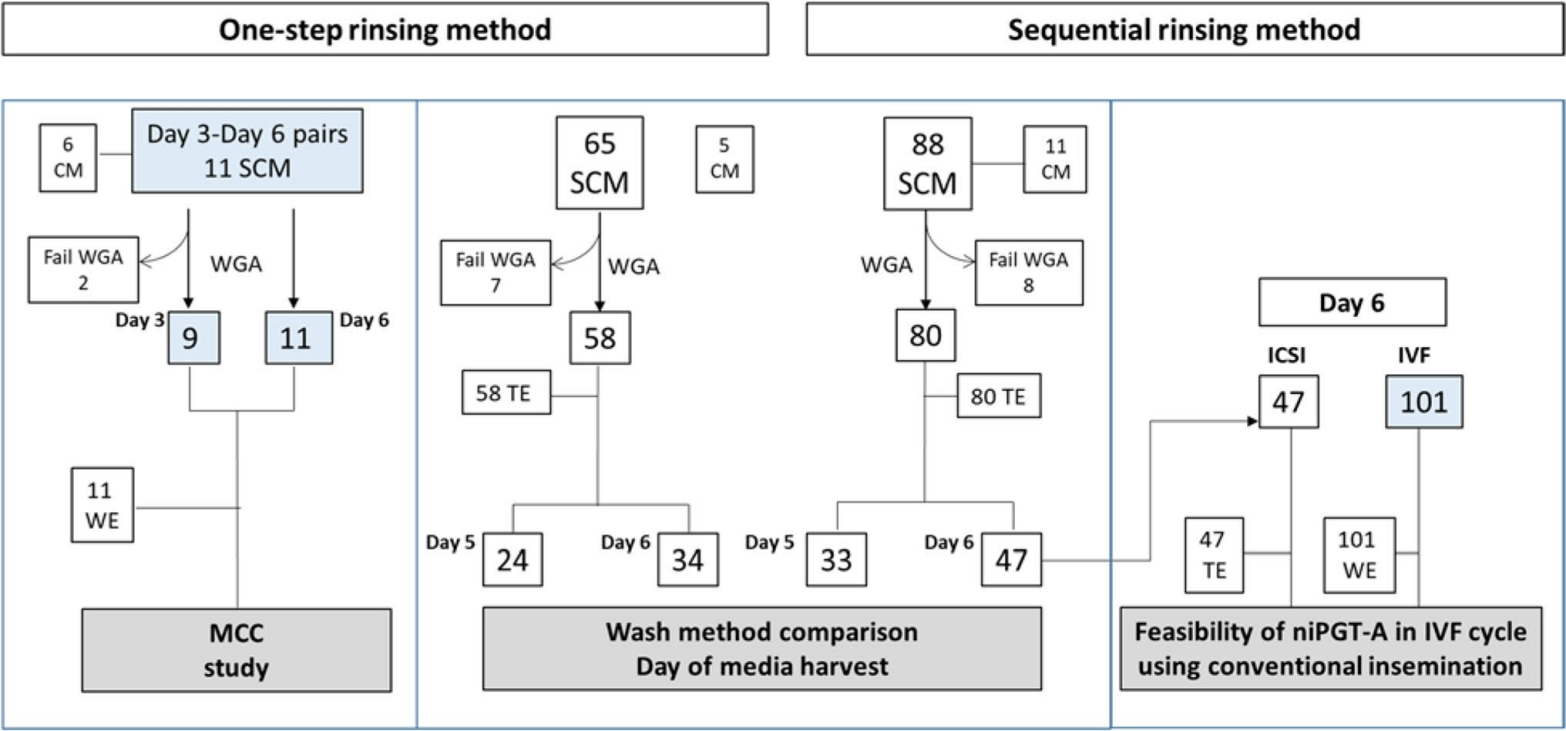
Flow chart of samples for the two different rinsing methods. SCM: spent culture media; CM: control media that put parallel to SCM but have no contact with embryo. WE: whole embryos; TE: trophectoderm biopsies; WGA: whole genome amplification; MCC: maternal cell/DNA contamination. SCM collected in IVF cycles using conventional insemination are highlighted in blue box

To determine the extent of maternal contamination of cfDNA in SCM during embryo culture, we analyzed 11 SCM pairs collected on day 3 and day 6 in the IVF cycles using conventional insemination (Fig. 1). There was no significant difference in amplification rate (9/11, 82.0% vs 11/11, 100%) and rate of informative results (9/9, 100% vs 9/11, 82.0%) between the SCM collected on day 3 and day 6 (Table 1). All control media showed no amplification. Their niPGT-A results were compared with the PGT-A results of the corresponding whole embryos. Concordance changed from 22.2% (2/9) in the day 3 SCM to 66.7% (6/9) in the day 6 samples, after one-step rinsing (Table 1). In fact, nine out of 11 embryos in this cohort were male, whereas all the results in the day 3 SCM were euploid female, indicating that the low concordance in the day 3 SCM was attributed by maternal cell / DNA contamination. Rinsing of embryos by one-step method before medium change on day 3 improved niPGT-A results by reducing maternal contamination. Analysis of the day 1 SCM (*n* = 14) resulted in 57.1% (8/14) of the samples with informative results. All of the results in day 1 SCM were euploid male, suggesting significant contamination by the inseminated sperm in the fertilization medium (Supplementary Table 1).

**Table 1:** niPGT-A in early window (days 1–3) of embryo culture. One step rinsing method was applied

	Day 3	Day 6
Amplification rate	81.8% (9/11)	100% (11/11)
Informative result	100% (9/9)	81.8% (9/11)
Total concordance with same sex	22.2% (2/9)	66.7% (6/9)
SCM shows 46,XX	100% (9/9)	22.2% (2/9)

### Comparison of the one-step rinsing and sequential rinsing methods

We analyzed 153 SCM from 153 individual embryos that were cultured from day 3 to the day of biopsy (day 5 or day 6) for PGT. The PGT-A results on the trophectoderm biopsy were used as references. Sixty-five embryos were rinsed by the one-step method, and 88 embryos were rinsed by the sequential method (Fig. 1). The proportion of day 5 samples were similar in both methods (sequential rinsing vs one-step rinsing, 44.3% vs 44.6% respectively, *p* = 0.97). All control media showed negative results after whole genome amplification (WGA). There was no significant difference in amplification rate (sequential rinsing vs one-step rinsing, 90.9% vs 89.2%, *p* = 0.73) and rate of informative result (sequential rinsing vs one-step rinsing, 81.3% vs 86.2%, *p* = 0.44) between the two rinsing methods.

In general, the rates of concordance were tended to be higher for the sequential rinsing than the one-step rinsing method (total concordance: sequential rinsing vs one-step rinsing, 75.4% vs 68.0%, *p* = 0.38; rate of total concordance with same sex: sequential rinsing vs one-step rinsing, 61.5% vs 50.0%, *p* = 0.22; Table 1A). A significant difference was observed in the rate of full concordance with same sex (sequential rinsing vs one-step rinsing, 47.7% vs 24.0%, *p* = 0.0092; Table 1A).

### Day of SCM collection

The rate of total concordance was significantly higher in day 6 samples than in day 5 samples, when the sequential rinsing method was applied (Table 2B, day 6 vs day 5, 85.0% vs 60.0%, *p* = 0.0228). Similar differences were observed in the rate of total concordance with same sex (day 6 vs day 5, 82.5% vs 28%, *p* < 0.0001) and in the rate of full concordance with same sex (day 6 vs day 5, 62.5% vs 24%, *p* = 0.0025).

**Table 2:** Comparison of two rinsing methods (A) and comparison of day of spent culture media collection (B), on the concordance rate of niPGT-A results. a: day 5 vs day 6 when sequential rinsing method was applied; b: one-step rinsing vs sequential rinsing when SCM was collected on day 6

	A			B				
				One-step		Sequential		
	One-step	Sequential	*P* value	Day 5	Day 6	Day 5	Day 6	*P* value
Total concordance	68.0%	75.4%	0.38	73.7%	64.5%^b^	60.0% ^a^	85.0% ^ab^	^a^ 0.0228 ^b^ 0.0449
Total concordance with same sex	50.0%	61.5%	0.22	42.1%	54.8%^b^	28.0% ^a^	82.5% ^ab^	^a^ < 0.0001 ^b^ 0.0113
Full concordance with same sex	24.0%	47.7%	0.0092	21.1%	25.8%^b^	24.0% ^a^	62.5% ^ab^	^a^ 0.0025 ^b^ 0.0021

The sequential rinsing method significantly out-performed the one-step rinsing method when the SCM were collected on day 6 instead of day 5. The rate of total concordance was higher in the day 6 samples using the sequential rinsing method (Table 2B; sequential rinsing vs one-step rinsing, 85% vs 64.5%, *p* = 0.0449). Differences were also observed in the rate of total concordance with same sex (sequential rinsing vs one-step rinsing, 82.5% vs 54.8%, *p* = 0.0113) and the rate of full concordance with same sex (sequential rinsing vs one-step rinsing, 62.5% vs 25.8%, *p* = 0.0021).

### Comparison of niPGT-A between IVF cycles using conventional insemination vs ICSI

There was no significant difference in terms of the rate of total concordance (conventional vs ICSI, 74.7% vs 85.0%, *p* = 0.19), the rate of total concordance with same sex (conventional vs ICSI, 71.6% vs 82.5%, *p* = 0.18), and the rate of full concordance with same sex (conventional vs ICSI, 61.1% vs 62.5%, *p* = 0.87) between media collected from embryo inseminated by the two methods (Table 3).

**Table 3:** Comparison of conventional insemination (IVF) vs intracytoplasmic sperm injection (ICSI) on the concordance rate of niPGT-A results

	ICSI	IVF	*P* value
Amplification rate	95.9% (47/49)	100% (101/101)	
Informative result	85.1% (40/47)	94.1% (95/101)	0.0732
Total concordance	85.0% (34/40)	74.7% (71/95)	0.1902
Total concordance with same sex	82.5% (33/40)	71.6% (68/95)	0.1819
Full concordance with same sex	62.5% (25/40)	61.1% (58/95)	0.8746

### Concordance by center

Using a well-defined sequential embryo rinsing protocol, consistent results of ni-PGTA was observed between the two centers (Supplementary Table 2). No significant difference in terms of the rate of total concordance (QMH vs KWH, 73.5% vs 75.4%, *p* = 0.84), the rate of total concordance with same sex (QMH vs KWH, 70.6% vs 72.1%, *p* = 0.87), and the rate of full concordance with same sex (QMH vs KWH, 55.9% vs 63.9%, *p* = 0.44).

## Discussion

This study evaluated two factors that may affect the performance of niPGT-A: embryo-rinsing method and day of SCM collection. We have shown a higher concordance rate when SCM was collected on day 6 after retrieval and the embryos were rinsed in a sequential manner when compared with conventional PGT-A using trophectoderm biopsy. We also demonstrated comparable results of niPGT-A when oocytes were fertilized by conventional insemination or ICSI.

Previous studies reported a concordance rate between PGT-A and niPGT-A ranging from 69.2 to 93.8% [22–24, 30, 31]. Such large variations in the concordance rate could be attributed to the different protocols used among studies. One of the major challenges of niPGT-A is maternal cell/DNA contamination that interferes the accuracy of the results [26]. Our data in day 3 SCM showed 100% euploid female but low concordance with whole embryos. This further suggested that contamination was mainly attributed to maternal DNA. It is crucial to exclude SCM from early culture period. In this study, embryos were transferred to fresh media on day 3 and the niPGT-A results in day 6 samples showed improvement in the concordance rate from 22.2 to 66.7%.

Early reports suggested the presence of maternal nuclear and organelle DNA in SCM [27] and the proportion of maternal contamination varied widely (0–100%) with a median percentage of embryonic DNA at approximately 8% [26]. To optimize the rinsing method, a sequential rinsing method was tested, resulting in an increase in the concordance rate. Such improvement was more remarkable when the SCM were collected on day 6 instead of day 5. Similar results was observed in a previous report in which the concordance rate in day 6/7 samples was significantly higher than those from day 5 samples (84.0% vs 63.0%) [31]. It was suggested that the amount of cfDNA accumulated with duration of embryo culture; therefore, the proportion of carry-over maternal DNA in SCM gradually reduced on the later days of embryo culture. It was reported that the number of samples having maternal cell/DNA contamination was dramatically reduced if SCM was harvested on day 6 or 7 versus day 5 [31].

Most of the published data on the concordance rate of niPGT-A were based on embryos from ICSI cycles in which cumulus cells on oocytes were enzymatically and mechanically removed before single sperm injection and cultured in fresh media [22–24, 31–33]. In IVF cycles using conventional insemination, oocyte-cumulus complexes were incubated with sperms for 16–18 h and zygotes were denuded mechanically on day 1 and transferred from the insemination drops to fresh media for culture. Our results indicated cfDNA of paternal origin in the day 1 SCM, but not in the day 6 SCM in this cohort. There were four sex discordant samples in the day 6 SCM collected in IVF cycles (Supplementary Table 3); all of them were contaminated with female DNA. Therefore, a higher chance of maternal cell/DNA contamination is expected in conventional insemination cycles. Consistently, we found that the percentage of successful amplification on day 3 samples was significantly higher in the conventional inseminated cycles (*n* = 11) than in the ICSI cycles (*n* = 17) (83.3% vs 11.8%, *p* < 0.0001, unpublished data). To extend the scope of niPGT-A, it is important to validate our niPGT-A protocol in IVF cycles using conventional insemination as ICSI is not indicated in infertile couples without severe male factor [34]. Our data showed that comparable niPGT-A results were obtained between conventional insemination and ICSI cycles.

A recent study on niPGT-A in IVF cycles reported similar ploidy concordance of 69.2% in the validation work and 75.0% in the clinical study when compared with trophectoderm biopsy [30]. However, our study was different in a few ways. First we used normally fertilized embryos (2PN) but not abnormal embryos (1PN) with which only 36.5% of them developed to blastocysts [30]. Second, only fresh embryos were included in our study but not a mixture of fresh and frozen embryos. Vitrification and warming process could enhance the release of embryonic cfDNA [35, 36] and may also effectively remove maternal cell/DNA contamination during embryo manipulation in these processes. In fact, the Xie’s study reported a significantly lower maternal contamination ratio in samples from cryopreserved blastocysts when compared with that of fresh blastocysts [30]. Finally, minimal modification on the IVF embryology protocol was involved in our study by adding a serial rinsing step without additional medium changes or extra cumulus cell removal procedure on day 4.

Multi-centre randomized trial allows quicker recruitment of the subjects from a wider population, and testing of the applicability of niPGT-A in a broader range of clinical settings, thus providing better basis for subsequent generalization of the findings. However, multi-centre randomized trial is more complex in terms of quality control and co-ordination. It is important to validate the performance of each centre by standardization of the laboratory protocol. Collection of non-utilizable embryos and SCM in IVF cycles using conventional insemination allows us to validate the performance of individual centers. This is especially useful when the centre has no experience in performing embryo biopsy. Our data showed that at a well-defined setting of embryo handling and day of SCM collection, a comparable concordance rate was observed between two studied centers. This is consistent with a recent study on 1301 embryos, reporting a concordance rate of 78.2% (ranged from 72.5–86.3%) involving eight centers [37].

In conclusion, we have shown a higher concordance rate when SCM was collected on day 6 after retrieval and the embryos were rinsed in a sequential manner when compared with conventional PGT-A using trophectoderm biopsy. Comparable results of niPGT-A when oocytes were fertilized by conventional insemination or ICSI. These optimization steps are important prior to commencement of a randomized trial in niPGT-A.

### Supplementary Information

Below is the link to the electronic supplementary material.

Supplementary file1 (DOCX 29 kb)

## Data Availability

The datasets used and/or analyzed during the current study are available from the corresponding author on reasonable request.
