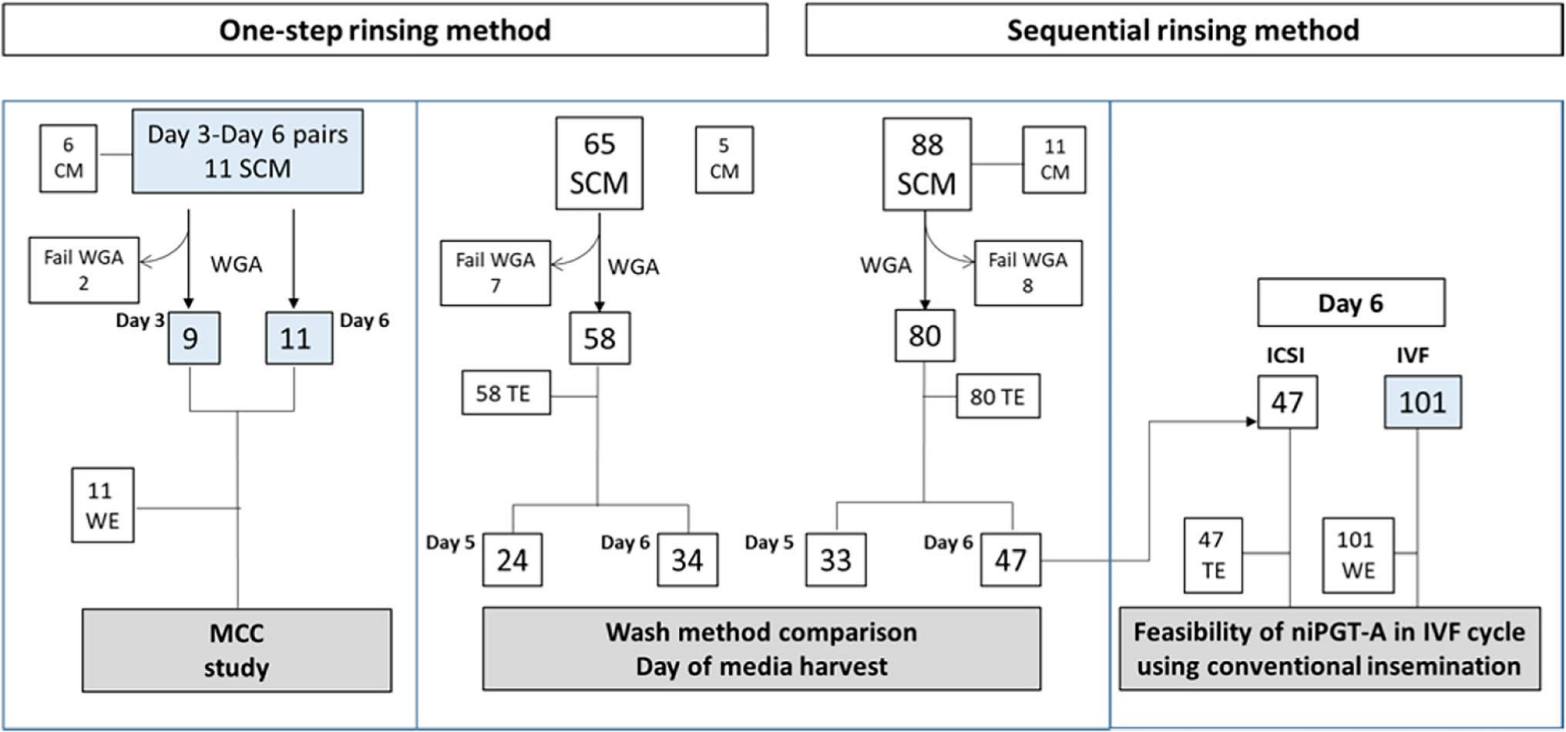# Correction to: Optimizing non‑invasive preimplantation genetic testing: investigating culture conditions, sample collection, and IVF treatment for improved non‑invasive PGT‑A results

**DOI:** 10.1007/s10815-024-03082-0

**Published:** 2024-03-07

**Authors:** Judy F. C. Chow, Kevin K. W. Lam, Heidi H. Y. Cheng, Shui Fan Lai, William S. B. Yeung, Ernest H. Y. Ng

**Affiliations:** 1 Department of Obstetrics and Gynaecology, School of Clinical Medicine, LKS Faculty of Medicine, The University of Hong Kong, Hong Kong, China; 2 Department of Obstetrics and Gynaecology, Queen Mary Hospital, Hong Kong, China; 3 Department of Obstetrics and Gynaecology, Kwong Wah Hospital, Hong Kong, China; 4 Shenzhen Key Laboratory of Fertility Regulation, The University of Hong Kong-Shenzhen Hospital, Shenzhen, China

**Correction to: Journal of Assisted Reproduction and Genetics**

10.1007/s10815-023-03015-3

In Figure 1 of this article, the data in column 3 should be IVF - 101 instead of IVF - 38; the figure image should have appeared as shown below in the second image.

The original article has been corrected.
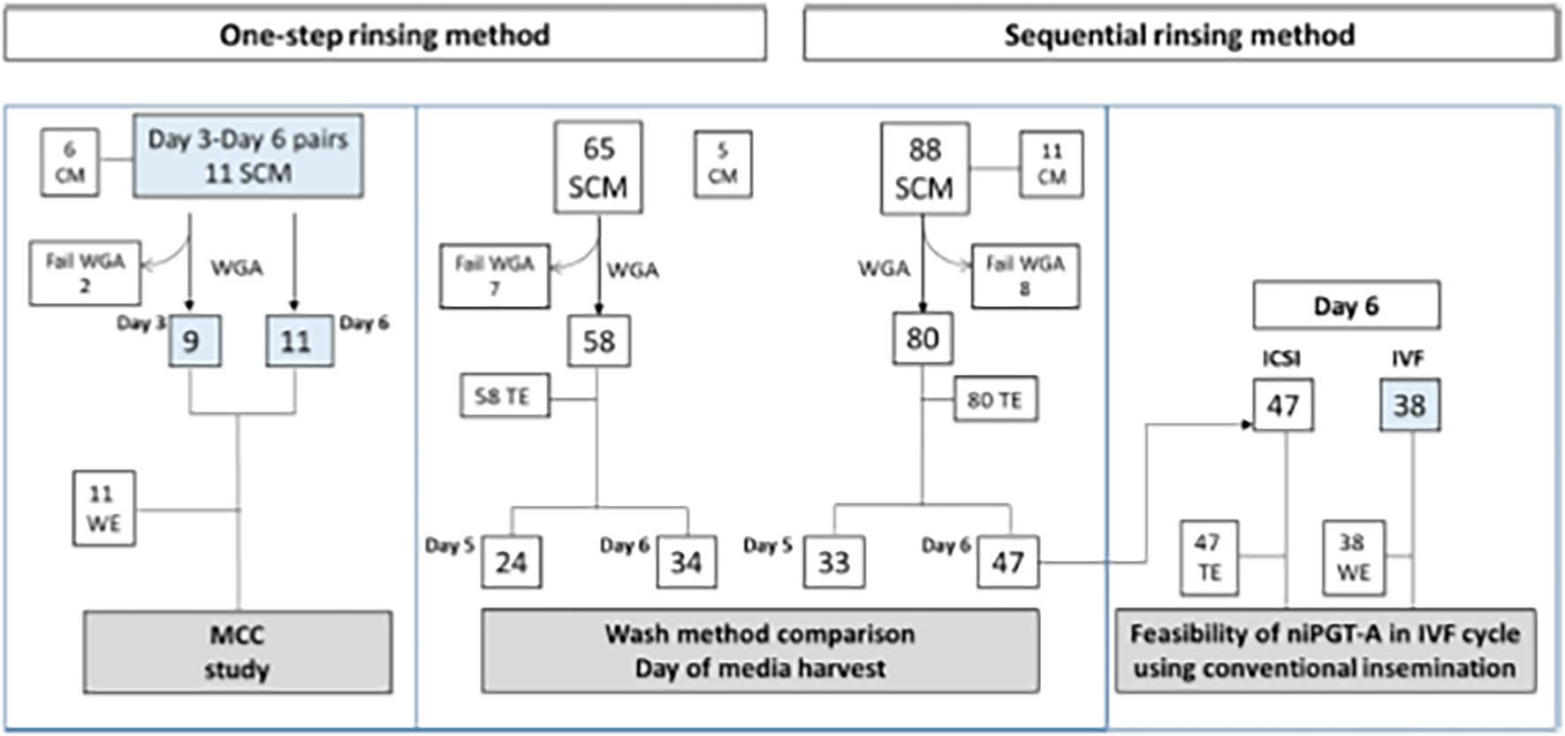


Correct Figure 1