# Supplementary material for: Optimizing non-invasive preimplantation genetic testing: investigating culture conditions, sample collection, and IVF treatment for improved non-invasive PGT-A results
Source: J Assist Reprod Genet. 2024 Jan 6;41(2):465–72. doi: 10.1007/s10815-023-03015-3 (PMC10894776; doi:10.1007/s10815-023-03015-3)
Supplement: Supplementary file 1 — Supplementary file1 (DOCX 29 kb) [file 10815_2023_3015_MOESM1_ESM.docx]

Supplementary Table 1: niPGT-A in insemination droplet (day 1) of embryo culture

|  | **Day 1** |
| --- | --- |
| Amplification rate | 100% (14/14) |
| Informative result | 57.1% (8/14) |
| SCM shows 46,XY | 100% (8/8) |

Supplementary Table 2: Comparison of niPGT-A results between two IVF centres

|  | Queen Mary Hospital | Kwong Wah Hospital | P value |
| --- | --- | --- | --- |
| Amplification rate | 100% (35/35) | 100% (66/66) |  |
| Informative result | 97.1% (34/35) | 92.4% (61/66) | 0.3398 |
| Total concordance | 73.5% (25/34) | 75.4% (46/61) | 0.8398 |
| Total concordance with same sex | 70.6% (24/34) | 72.1% (44/61) | 0.8730 |
| Full concordance with same sex | 55.9% (19/34) | 63.9% (39/61) | 0.4404 |

| Supplementary Table 3: Concordance of niPGT-A in IVF cycles, with discordant sex | | | | | | | | | | | |
| --- | --- | --- | --- | --- | --- | --- | --- | --- | --- | --- | --- |
|  |  | **Poor quality embryo (PQE)** | |  |  | | **Spent culture media (SCM)** | | |  | |
| Study ID | Embryo Code | Gender | Result | SCM code | | Gender | | Result | | | Concordance |
| ni1020 | 9 | Male | No aneuploidy detected | SM1113 | | Female | | | No aneuploidy detected | | C |
| ni1042 | 2 | Male | No aneuploidy detected | SCMK023 | | Female | | | No aneuploidy detected | | C |
| ni1056 | 13 | Male | No aneuploidy detected | SCMK054 | | Female | | | No aneuploidy detected | | C |
| ni1064 | 10 | Male | Abnormal; +1, +13, +21 | SCMK086 | | Female | | | No aneuploidy detected | | D |
|  |  |  |  |  |  | |  | | | |  |
|  |  |  |  |  | C | | Total concordant | | |  | |
|  |  |  |  |  | D | | Discordant | | |  | |
